# Tick transmission of *Borrelia burgdorferi* to the murine host is not influenced by environmentally acquired midgut microbiota

**DOI:** 10.1186/s40168-022-01378-w

**Published:** 2022-10-17

**Authors:** Sukanya Narasimhan, Nallakkandi Rajeevan, Morven Graham, Ming-Jie Wu, Kathleen DePonte, Solenne Marion, Orlanne Masson, Anya J. O’Neal, Joao H. F. Pedra, Daniel E. Sonenshine, Erol Fikrig

**Affiliations:** 1 Department of Internal Medicine, Section of Infectious Diseases, New Haven, USA; 2 Yale Centre for Molecular Informatics, New Haven, USA; 3 Department of Cell Biology, Yale University School of Medicine, New Haven, CT 06420 USA; 4 Current address: Roche Diagnostics International, 6343 Rotkreuz, Switzerland; 5 Department of Microbiology and Immunology, University of Maryland School of Medicine, Baltimore, MD 21201 USA; 6 Laboratory of Malaria and Vector Research, National Institute of Allergy and Infectious Diseases, Rockville, MD 20852 USA

**Keywords:** *Ixodes scapularis*, Tick microbiome, Tick innate immunity, Transmission, *Borrelia burgdorferi*

## Abstract

**Background:**

*Ixodes scapularis* is the predominant tick vector of *Borrelia burgdorferi*, the agent of Lyme disease, in the USA. Molecular interactions between the tick and *B. burgdorferi* orchestrate the migration of spirochetes from the midgut to the salivary glands—critical steps that precede transmission to the vertebrate host. Over the last decade, research efforts have invoked a potential role for the tick microbiome in modulating tick-pathogen interactions.

**Results:**

Using multiple strategies to perturb the microbiome composition of *B. burgdorferi*-infected nymphal ticks, we observe that changes in the microbiome composition do not significantly influence *B. burgdorferi* migration from the midgut, invasion of salivary glands, or transmission to the murine host. We also show that within 24 and 48 h of the onset of tick feeding, *B. burgdorferi* spirochetes are within the peritrophic matrix and epithelial cells of the midgut in preparation for exit from the midgut.

**Conclusions:**

This study highlights two aspects of tick-spirochete interactions: (1) environmental bacteria associated with the tick do not influence spirochete transmission to the mammalian host and (2) the spirochete may utilize an intracellular exit route during migration from the midgut to the salivary glands, a strategy that may allow the spirochete to distance itself from microbiota in the midgut lumen effectively. This may explain in part, the inability of environment-acquired midgut microbiota to significantly influence spirochete transmission. Unraveling a molecular understanding of this exit strategy will be critical to gain new insights into the biology of the spirochete and the tick.

Video Abstract

**Supplementary Information:**

The online version contains supplementary material available at 10.1186/s40168-022-01378-w.

## Introduction

*Ixodes scapularis*, the black-legged tick, is widely distributed in the eastern United States with geographic expansion occurring northward towards Canada and to the southern Midwest [1–3]. *I. scapularis* vectors’ multiple human pathogens include *Borrelia burgdorferi*, the agent of Lyme disease [4]. Proteomic, genetic, and transcriptomic studies over the last few decades demonstrate that *B. burgdorferi*, and the tick engages in molecular interactions critical for spirochete colonization of the tick and for spirochete transmission to the vertebrate host [5, 6]. Studies over the last few years have suggested that ticks are exposed to diverse environmental microbiota during their lifecycle and that this microbiota may additionally influence tick-spirochete interactions [7, 8]. While nutritional endosymbionts may be acquired transovarially from the mother (e.g., *Rickettsia buchneri*), environmental microbiota may be horizontally acquired during different stages of the tick life cycle [8]. There is consensus that the *Ixodes* microbiome is composed predominantly of the vertically transmitted *Rickettsial* endosymbiont, a putative nutritional symbiont, and that the commensal microbiota that the tick acquires from the environment is variable [8, 9]. Depending on the development stage, age, geographic location, and host utilized for tick feeding, the complexity and diversity in tick microbiome compositions vary [10–13] and present a confounding element in tick microbiome research. Suboptimal processing of tick samples has also been suggested to result in the inclusion of surface contaminants that artificially inflate the complexity of the tick microbiome. Taking these factors into consideration, Ross et al. have suggested that the tick microbiome is simple, transient, and not stable [14]. Studies also indicate that tick-microbiota associations, even if transient, may impact tick biology and vectorial competence [15–19].

Our earlier work suggested that when an uninfected *I. scapularis* tick feeds on a *B. burgdorferi*-infected vertebrate host, the spirochete enters the tick and *I. scapularis*-associated microbiota impact tick midgut colonization of *B. burgdorferi* [16, 20]. These findings provided the impetus to determine whether tick-associated environmental microbiota may also play a role in spirochete transmission from the tick to the vertebrate host. We bear in mind that spirochete entry into the tick midgut and exit from the midgut are fundamentally different events modulated by multiple factors that are not fully understood [5]. In this study, we present observations that suggest that changes in the tick midgut bacterial microbiome do not significantly impact *B. burgdorferi* exit from the midgut and transmission to the vertebrate host. Our observations also draw attention to a novel facet of tick-spirochete interactions in the midgut that may explain in part the inability of environmental microbiota to influence spirochete transmission.

## Results

### Impact of *B. burgdorferi* colonization on the tick midgut microbiome composition

**Fig. 1:**
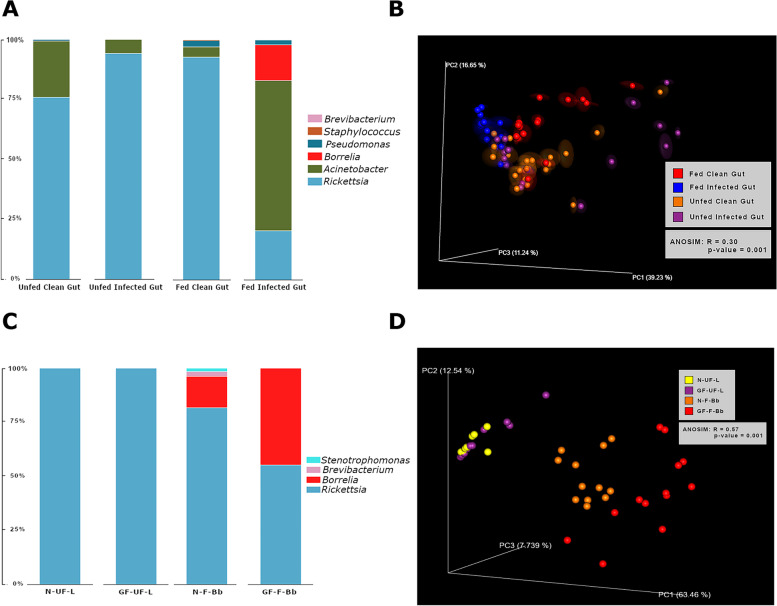
Microbiome composition of *I. scapularis* nymphs and larvae. 16S rRNA sequence analysis of microbial communities of **A** predominant bacterial genera in the midguts of flat uninfected (unfed clean) or *B. burgdorferi*-infected (unfed infected) and fed uninfected (fed clean) or *B. burgdorferi*-infected (fed infected) nymphs. **B** Principal coordinate analysis of weighted UniFrac distances of microbial communities between the groups. **C** Predominant bacterial genera in unfed larvae raised in normal incubators (N-UF-L) or in germ-free isolators (GF-UF-L) or in fed *B. burgdorferi*-infected larvae raised in a normal incubator (N-F-Bb) or in germ-free isolate (GF-F-Bb). **D** Principal coordinate analysis of weighted UniFrac distances of microbial communities between the groups. Statistical significance determined by ANOSIM is indicated within each plot in **B** and **D**. Each data point in **A** and **B** represents a pool of 5 unfed nymphal midguts or individual fed nymphal midgut. Each data point in **B** and **C** represents a pool of 50 unfed larvae or 5 fed larvae

The tick genome encodes innate immune signaling pathways potentially capable of engaging with Gram-positive and Gram-negative bacteria and eliciting antimicrobial responses [21]. Whether *B. burgdorferi* colonization of the tick modulates tick innate immune responses and whether this in turn influences the tick microbiome composition is not understood. To address this, we dissected midguts from unfed or fed uninfected or *B. burgdorferi*-infected nymphs and processed the midguts for DNA purification and 16S rRNA (ribosomal RNA) amplicon sequencing as described in the “Materials and methods” section. The sequences were filtered, and phylogenetic analysis was conducted using the FastTree computational tool [22]. Taxa present at less than 1 % relative abundance in all samples was excluded from the analysis to preclude bias towards potential environmental contaminants. We observed that *Rickettsia* was the predominant genera in unfed nymphal ticks regardless of infection status. While *Rickettsia* was also predominant in repleted nymphal ticks, the relative abundance of *Acinetobacter* species was increased in *B. burgdorferi*-infected fed nymphal guts when compared to that in fed clean nymphal guts. The differences in the composition of the microbiome between unfed and fed *B. burgdorferi*-infected and uninfected nymphal midguts (Fig. 1A) were limited to differences in the relative abundance of *Acinetobacter, Staphylococcus, Brevibacterium*, and *Pseudomonas* genera, potentially associated with ticks from the host skin or environment. Qualitative and quantitative distance metrics were computed to derive the beta diversity. Principal coordinate analysis (PCoA) showed significant differences in diversity between unfed and fed uninfected and infected nymphs (Fig. 1B).

### Microbiome composition of ticks raised in germ-free or normal incubators

Off-host phases of the tick life cycle are spent under leaf litter and in soil and offer ample opportunities to encounter and associate with environmental bacteria. On-host phases during bloodmeal acquisition also expose the tick to commensal microbiota in the host skin [23]. Our earlier work showed that larval ticks raised in sterile containers had a microbiome composition different from that of larvae raised in normal containers [16]. Since larvae raised in sterile containers had to be fed on a murine host to progress to the nymphal stage, multiple exposures to the normal environment precluded successful maintenance of dysbiosed nymphs using the sterile container method. Therefore, we sought to generate larval ticks in germ-free isolators as described in the “Materials and methods” section. Briefly, mated and engorged female adults were surface sterilized and transported into a germ-free isolator and maintained in humidified glass desiccators. Control adults were similarly maintained in normal incubators under comparable humidity and temperature conditions. Time to egg laying, egg mass (by visual inspection), and larval molting were comparable in germ-free and normal conditions.

The microbiome composition of whole larval ticks was assessed by 16S amplicon sequencing as outlined in the “Materials and methods” section. Any taxa present at less than 1 % relative abundance in all samples was excluded from the analysis to preclude potential bias towards environmental contaminants. Using these analysis parameters, the microbiome composition of unfed larvae raised in germ-free isolators was not significantly different from that of larvae raised in normal incubators and was predominantly composed of the *Rickettsial* endosymbiont (Fig. 1C). The germ-free-raised (GF) larvae were then allowed to feed to repletion on germ-free C57/BL6 mice infected with *B. burgdorferi* as described in the “Materials and methods” section. Control larvae were similarly fed on *B. burgdorferi*-infected pathogen-free C57/BL6 mice, and the microbiome composition of fed larval ticks was assessed as described for unfed larval ticks. The microbiome composition of fed GF and normal larvae was also dominated by *Rickettsial* species (Fig. 1C), although fed GF larvae had decreased relative abundance of *Rickettsial* bacteria and increased relative abundance of *Borrelia*. While GF larvae were not associated with any other bacterial genera other than *Borrelia* and *Rickettsia,* normal incubator-raised larvae were also associated with genera such as *Brevibacterium* and *Stenotrophomonas*, representing environmental bacteria. PCoA analysis to assess beta diversity of the microbiome revealed significant differences between fed GF and normal-raised larvae and not between unfed GF and normal-raised larvae (Fig. 1D).

### *B. burgdorferi* growth and migration in the tick and transmission to the murine host are not impacted in nymphs raised in germ-free isolators

**Fig. 2:**
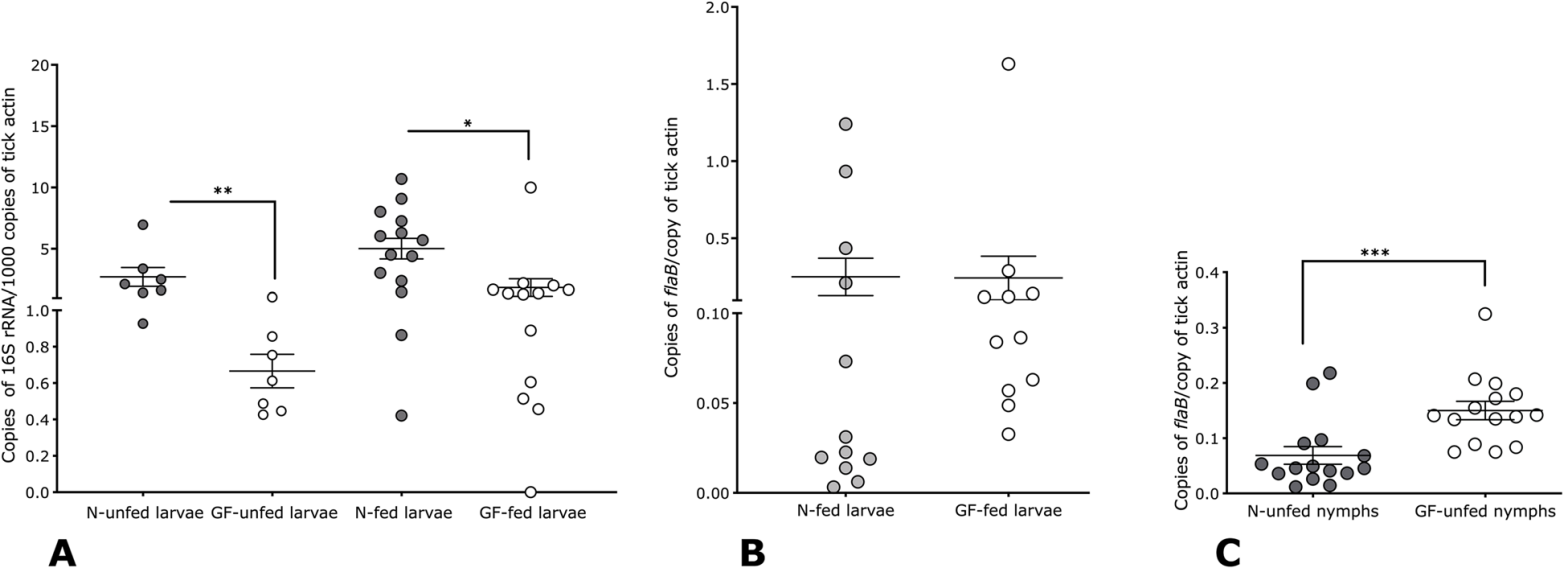
*Borrelia burgdorferi* colonization is altered in germ-free isolator-raised *Ixodes scapularis* larvae. Quantitative PCR evaluation of **A** 16S rRNA amplicon as a measure of total bacterial abundance in unfed *I. scapularis* larvae raised in normal (N-unfed) or in germ-free (GF-unfed) isolators and in *I. scapularis* larvae raised in normal incubators and fed on *B. burgdorferi*-infected C57/B6 mice (N-fed) or *I. scapularis* larvae raised in germ-free isolators and fed on germ-free *B. burgdorferi*-infected C57/B6 mice (GF-fed). **B***FlagellinB* (*flaB*) abundance in *I. scapularis* larvae raised in normal incubators and fed on *B. burgdorferi*-infected C57/B6 mice (N-fed); *I. scapularis* larvae raised in germ-free isolators and fed on germ-free *B. burgdorferi*-infected C57/B6 mice (GF-fed). **C**. *B. burgdorferi* burden in molted unfed *I. scapularis* nymphs raised in normal (N-unfed) or in germ-free (GF-unfed) isolators. Error bars are + SEM. Significance of differences assessed by non-parametric Mann-Whitney test (*p< 0.05, **p<0.005, ***p < 0.0001). Each data point in **A** and **B** represents a pool of 50 unfed larvae or 5 fed larvae. Each data point in C represents a pool of 5 unfed nymphal midguts

To further validate the global observations on the microbiome composition, we assessed the total bacterial burden in unfed larvae by quantitative PCR (qPCR). Unfed GF larvae had significantly diminished total bacterial burden compared to normal larval ticks as seen by qPCR using 16S universal primers as a proxy for bacterial presence (Fig. 2A). Further, upon feeding germ-free larvae on germ-free C57/BL6 mice, we also observed significantly decreased total bacteria compared to fed normal larvae as seen by qPCR using 16S universal primers as a proxy for bacterial presence (Fig. 2A). GF larvae fed on *B. burgdorferi*-infected germ-free C57/BL6 mice acquired *B. burgdorferi* burden comparable to normal larvae fed on normal mice as seen by qPCR using *B. burgdorferi*-specific *flagellinB* (*flaB*) as a proxy for *B. burgdorferi* presence (Fig. 2B). However, fed germ-free larvae molted to nymphs in the germ-free isolator showed significantly higher *B. burgdorferi* burden as seen by qPCR detection of *flaB* amplicons suggesting a favorable environment for spirochete survival during the molt (Fig. 2C).

Time to molting to the nymphal stage was comparable in both groups-approximately 6–8 weeks. Despite comparable total bacterial burden in nymphs molted in normal or GF isolators as seen by qPCR detection of 16S rRNA amplicons in nymphal midguts (Supplemental Fig 1A), the microbiome composition was different between GF-raised and normal nymphs as seen by qPCR assessment of specific predominant genera frequently associated with *I. scapularis* ticks (Supplemental Fig 1). We have in this study focused on *Staphylococcus* spp., as an example of Gram-positive commensal bacteria found on mammalian skin [24] and on *Pseudomonas* spp., as an example of Gram-negative bacteria found in soil and also frequently associated with ticks [8]. We observed a significant decrease in the abundance of *Staphylococcus* spp. in GF-isolator-raised nymphs compared to that in normal isolator-raised nymphs (Supplemental Figure 1C). The abundance of *Pseudomonas* spp. also trended to be less in GF-isolator-raised nymphs when compared to that in normal isolator-raised nymphs (Supplemental Figure 1D). A similar assessment of GF or normal isolator nymphs fed on GF-or normal C57/BL6 mice respectively showed comparable total bacterial abundance but a significant decrease in abundance of *Pseudomonas* spp. in GF-isolator-raised nymphs when compared to that in normal isolator-raised nymphs (Supplemental Figure 1E and H).

**Fig. 3:**
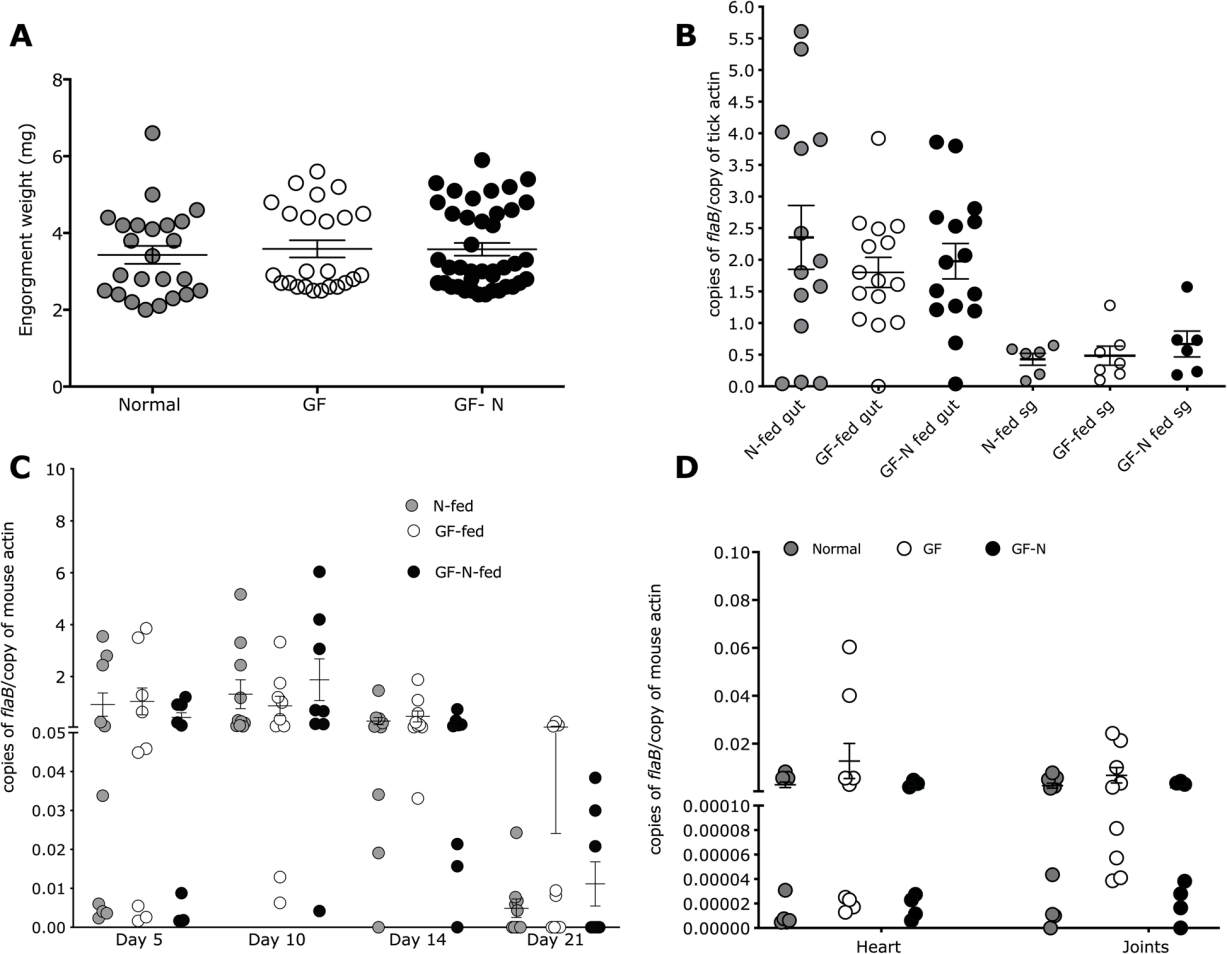
*Borrelia burgdorferi* migration in ticks and transmission to the murine host is not altered in germ-free isolator-raised nymphs. *B. burgdorferi*-infected nymphal ticks were raised in a normal incubator and fed on pathogen-free C57/BL6 mice (N) or raised in germ-free isolators and fed on germ-free C57/BL6 mice (GF) or raised in germ-free isolators and fed on pathogen-free C57/BL6 mice (GF-N) and the following parameters were assessed: **A** Engorgement weights; qPCR assessment of **B***B. burgdorferi* burden in midguts (gut) and salivary glands (sg); **C***B. burgdorferi* burden in the murine skin at days 5, 10, 14, and 21 post-tick detachment; and **D***B. burgdorferi* burden in the murine hearts and joints 21 days post-tick detachment. Error bars are + SEM. The significance of differences was assessed by ANOVA with Dunn’s multiple comparison test. Each data point in **A** is a single tick, in **B** is a pool of 2–3 tick midguts or salivary glands, and in **C** and **D** a single mouse

To assess the efficiency of transmission by *B. burgdorferi*-infected germ-free or control nymphs, 3–4 GF or control nymphs were then fed on each of ten germ-free or pathogen-free C57/BL6 mice. GF nymphs were also fed on normal pathogen-free C57/BL6 mice to rule out the impact of differential immune responses inherent to germ-free mice [25] on the outcomes. The engorgement weights of ticks were comparable between normal and GF isolator-raised nymphs fed on normal or germ-free mice (Fig. 3A). Despite changes in the microbiome composition of normal and GF-nymphs (Supplemental Figure 1), *B. burgdorferi* burden as seen by qPCR detection of *flaB* amplicons was comparable between normal and GF-raised repleted nymphs in the midguts and salivary glands (Fig. 3B). Transmission to the murine host was also comparable as seen by qPCR assessment of *B. burgdorferi* burden in the skin of mice fed upon by germ-free or normal nymphal ticks on 5, 10, 15, and 21 days post-tick detachment (Fig. 3C). Dissemination to distal tissues including the heart and joints were also comparable in all three groups (Fig. 3D).

### Dysbiosis by antibiotic treatment does not influence spirochete growth and migration in the tick

**Fig. 4:**
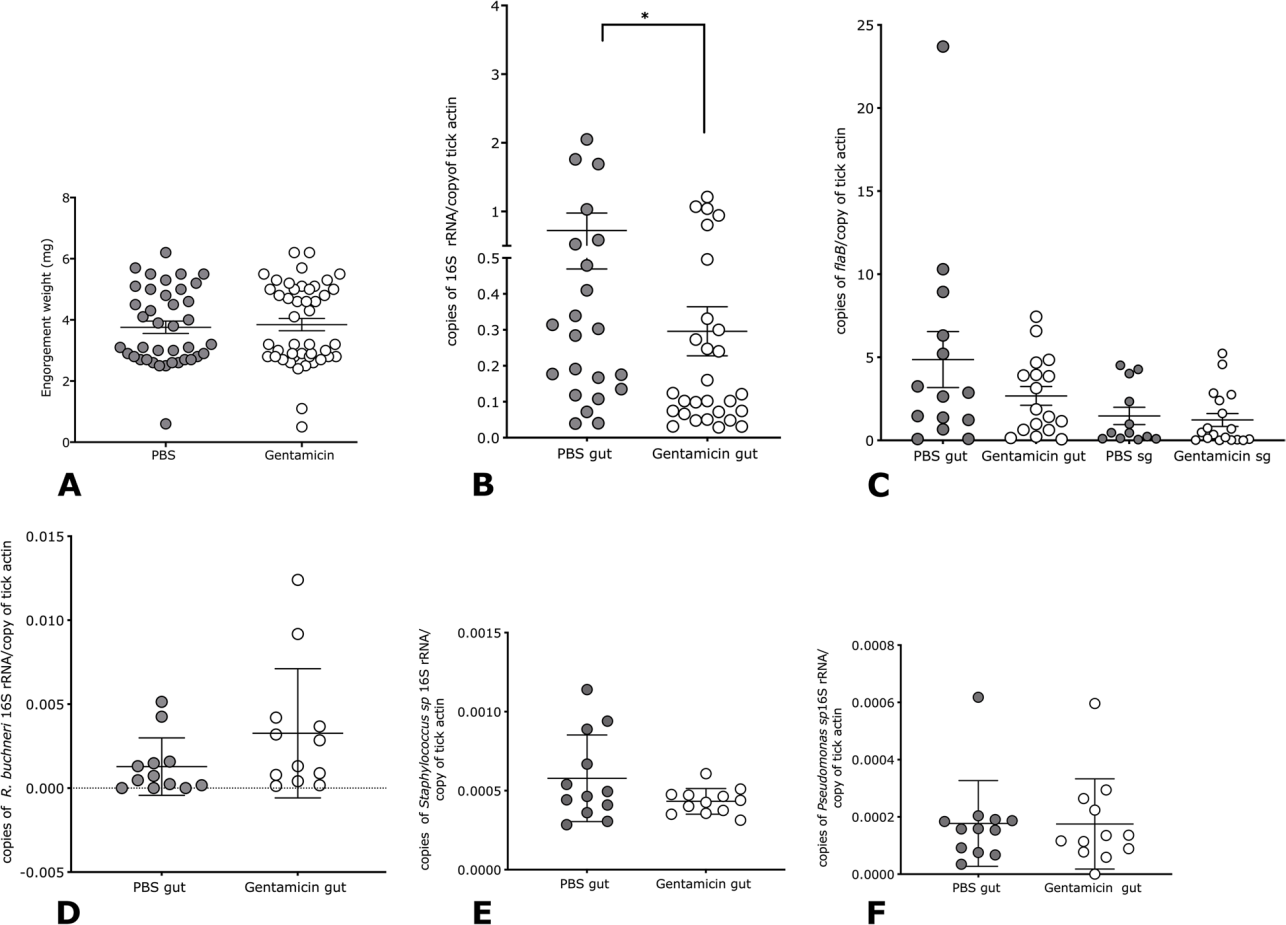
*Borrelia burgdorferi* growth in the midgut and migration to the salivary glands is not impaired in gentamicin-treated *B. burgdorferi*-infected nymphal ticks. GFP (green fluorescent protein)-transgenic *B. burgdorferi* (297)*-*infected nymphs were fed to repletion on gentamicin-treated-C3H/HeN mice and the following parameters were assessed: **A** Engorgement weights, qPCR assessment of **B** total bacterial burden, **C***B. burgdorferi* burden in midguts and salivary glands, **D***Rickettsia buchneri (R. buchneri)-*specific 16S rRNA, **E***Staphylococcus genera-*specific 16S rRNA, and **F***Pseudomonas genera-*specific 16S rRNA*.* Each data point in **A** represents a single tick and in all other panels, a pool of 3 ticks. Error bars are + SEM. Significance of differences assessed by non-parametric Mann-Whitney test (*p<0.05)

Our earlier study utilized gentamicin to generate dysbiosed larval ticks [16]. We therefore used the same strategy to perturb the microbiome composition of *B. burgdorferi-*infected nymphal ticks. We generated GFP-gentamicin-transgenic *B. burgdorferi,* Bb914 (297 strain)-infected nymphal ticks as described earlier [26]. The mice were injected intraperitoneally with gentamicin a day before the nymphal tick challenge and on the day of the tick challenge as described in the “Materials and methods” section to allow circulating gentamicin to enter the tick midgut along with the bloodmeal. It must be noted that gentamicin would act against a broad spectrum of environmental bacteria associated with the tick but would not be bactericidal to gentamicin-transgenic *Bb914.* Control mice were similarly injected with PBS. Ticks fed to repletion and engorged comparably on PBS or gentamicin-injected mice (Fig. 4A). Total bacterial burden was, expectedly, significantly reduced in gentamicin-exposed ticks (Fig. 4B). However, *B. burgdorferi* growth in the midgut and migration to the salivary glands was not affected (Fig. 4C) as seen by qPCR assessment of spirochete burden. qPCR assessment showed modest changes in abundance of *R. buchneri* and of *Staphylococcus* spp. (Fig. 4D, E) indicating an impact of gentamicin on the microbiome. However, no change in the abundance of *Pseudomonas* spp. was observed (Fig. 4F).

### RNAi-mediated silencing of signal transducer and activator of transcription (stat) alters the midgut microbiome composition but does not impact spirochete migration in the tick and transmission to the murine host

**Fig. 5:**
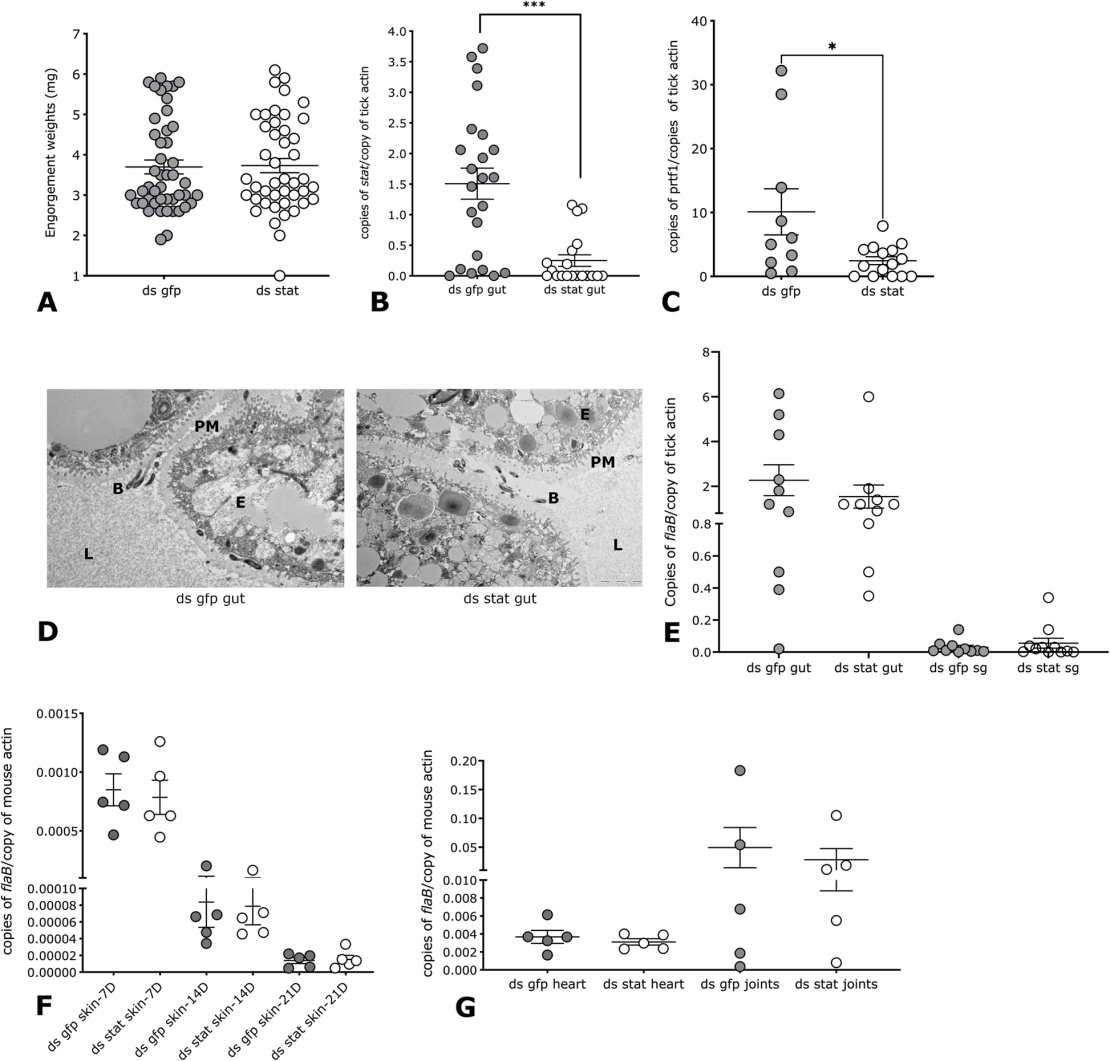
Decreasing the expression of signal transducer and activator of transcription (stat) does not impact spirochete migration in the tick and transmission to the murine host. Double-stranded *stat* RNA (ds stat) or control ds gfp RNA (ds gfp) was injected into the anal pore of *B. burgdorferi*-infected nymphs and ticks fed to repletion on pathogen-free C3H/HeN mice and the following parameters evaluated: **A** Engorgement weights; qRT-PCR assessment of transcript levels for **B***stat* in the tick midguts; **C** peritrophin1 (prtf1) in tick midguts; **D** transmission electron microscopy of 48-h-fed midguts; qPCR assessment of *B. burgdorferi* burden in **E***flaB* in the tick midguts and salivary glands (sg); **D** Skin of mice at days 7, 14, and 21 days post-tick detachment; and **E** the heart and joints of mice at 21 days post-tick detachment. Each data point in **A** represents 1 tick; **B**, **C**, and **E** represent a pool of 3 ticks; and **F** and **G** represent one mouse. Error bars are + SEM. Significance of differences assessed by non-parametric Mann-Whitney test in **A**, **B**, **C**, **E**, **F**, and **G** (***p<0.0001)

The tick genome encodes the predominant components of the Janus kinase (JAK)/signal transducer and activator of transcription (STAT) pathway [27], an evolutionarily conserved signaling pathway invoked in repair and remodeling of the midgut epithelial cells and in activating immune responses in arthropods [28, 29]. Our earlier work suggested that the microbiome modulates the JAK/STAT pathway of the tick and influences the integrity of the peritrophic matrix (PM) and this has a functional consequence on spirochete colonization of the midgut [16]. As a corollary, we reasoned that decreasing the expression of *stat* by RNA interference would have an impact on midgut PM integrity, immune responses, and consequently the microbiome. Therefore, to determine if PM integrity and microbiome were also critical during spirochete transmission to the murine host, we knocked down the expression of *stat* by anal pore injection of ds (double-stranded) *stat* RNA into *B. burgdorferi*-infected nymphs as described in the “Materials and methods” section. Control ticks were similarly injected with ds gfp (green fluorescent protein) RNA, and ds stat or ds gfp-injected nymphs were allowed to engorge on pathogen-free C3H/HeN mice. Both groups engorged comparably (Fig. 5A). Salivary glands and midguts were dissected from engorged ticks and processed for qRT(reverse transcriptase)-PCR as described in the “Materials and methods” section. We observed a significant decrease in *stat* expression as seen by the qRT-PCR assessment of *stat* transcripts (Fig. 5B). Consistent with our earlier observation that Stat regulates the expression of Peritrophin-1, a key component of the PM [16], the expression of *peritrophin 1* was significantly reduced (Fig. 5C). The transmission electron microscopic assessment of ds gfp or ds stat RNA-injected tick midguts 48 h post-tick feeding also showed that the PM of *stat-*knockdown ticks was less electron-dense compared to that in control midguts (Fig. 5D), indicative of a compromised PM. However, *stat*-knockdown ticks had no impact on *B. burgdorferi* growth in the tick midgut and on the invasion of salivary glands as seen by comparable *flaB* transcripts in control and knockdown nymphs assessed by qPCR (Fig. 5E).

Total bacterial burden as seen by qPCR of 16S rRNA amplicon was comparable in ds stat-or ds gfp-injected ticks (Supplemental Fig 2A). The abundance of the endosymbiont *R. buchneri* [30] was increased in stat-knockdown nymphal midguts (Supplemental Fig 2B). While the abundance of Gram-negative bacteria of the genera *Pseudomonas* was not influenced, Gram-positive commensal bacteria of the genera *Staphylococcus* were significantly decreased in *stat* knockdown nymphal midguts (Supplemental Fig 2C-D).

To assess the efficiency of transmission by *B. burgdorferi*-infected stat-knockdown nymphs, 3–4 stat-knockdown or control nymphs were then allowed to engorge on each of 5 pathogen-free C3H/HeN mice. Transmission to the murine host was comparable as seen by qPCR assessment of *B. burgdorferi* burden in the skin of mice fed upon by stat knockdown or control nymphal ticks at 7, 14, and 21 days post-tick detachment (Fig. 5F). Dissemination to distal tissues including the heart and joints were also comparable in both groups (Fig. 5G).

### RNAi-mediated silencing of xiap and p47 impacts *B. burgdorferi* growth in the tick midgut without impacting migration to the salivary glands

**Fig. 6:**
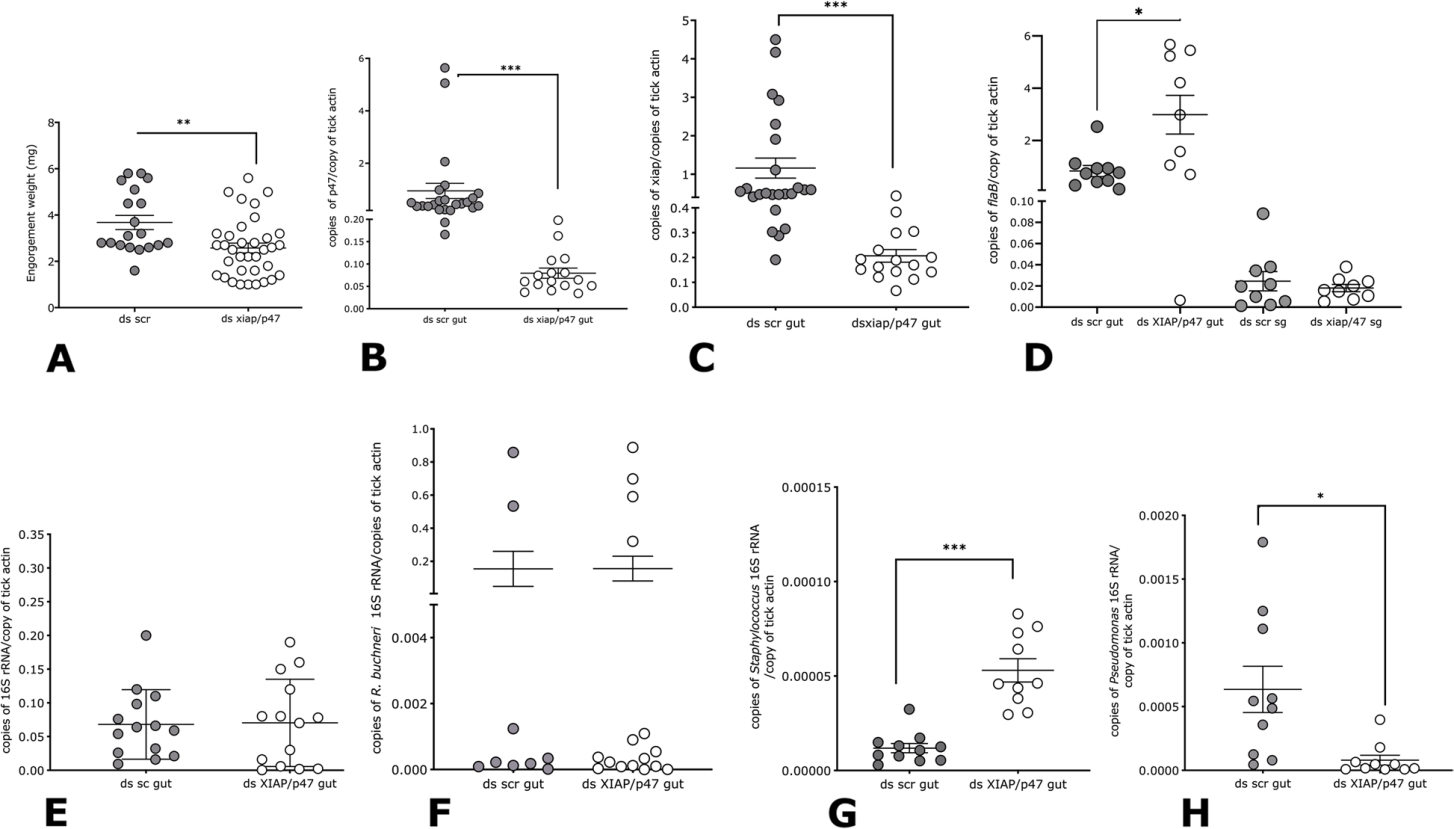
Impairing key regulators of the immune deficiency pathway (IMD) influences spirochete growth but not migration in the tick. A cocktail of short interfering (si) RNA targeting key components of the IMD pathway, XIAP and p47 (ds xiap/p47), or control ds gfp RNA (ds gfp) was injected into the anal pore of *B. burgdorferi*-infected nymphs and ticks fed to repletion on pathogen-free C3H/HeN mice and the following parameters evaluated: **A** Engorgement weights; qRT-PCR assessment of transcript levels for **B** p47 in tick midguts; **C** xiap in tick midguts; qPCR assessment of **D***B. burgdorferi*-specific *flagellin B* (flaB) in tick midguts and salivary glands (sg); E 16S rRNA as a proxy for total bacterial burden; **F***Rickettsia buchneri (R. buchneri)-*specific 16S rRNA; **G***Staphylococcus genera-*specific 16S rRNA; **H***Pseudomonas genera-*specific 16S rRNA*.* (sg); Each data point in **A** represents 1 tick; **B–H** represent a pool of 3 ticks; Error bars are + SEM. Significance of differences assessed by non-parametric Mann-Whitney test (***p<0.0001; *p<0.05)

Shaw et al. [31] showed that despite lacking several components of the IMD (immune deficiency), the IMD pathway in *Ixodes* is functional. Whether impairing key activation components of this pathway such as XIAP (X-linked inhibitor of apoptosis) and p47, the binding partner of XIAP that induces a signaling cascade within the IMD pathway [32] would impact the microbiome composition and *B. burgdorferi* growth during transmission is not known. Towards this, using the RNAi approach, we knocked down the expressions of *xiap* and *p47.* Subsequently, we assessed tick feeding and *B. burgdorferi* growth and migration to the salivary glands. Ticks microinjected with a cocktail of ds xiap and p47 showed a significant impairment of tick feeding compared to ticks injected with ds gfp RNA as seen by engorgement weights (Fig. 6A). Both *xiap* and *p47* transcripts were significantly reduced in ds xiap/p47-injected ticks compared to that in ds gfp-injected ticks as determined by qRT-PCR assessment of knockdown efficiency (Fig. 6B, C). qPCR assessment showed significantly increased *B. burgdorferi* burden in ds xiap/p47-injected nymphal midguts, although migration to the salivary glands was not impacted (Fig. 6D). The total bacterial abundance as seen by qPCR assessment showed comparable abundance in both groups (Fig. 6E). While the abundance of the endosymbiont *R. buchneri* (Fig. 6F) was comparable between the two groups, a significant increase in the abundance of *Staphylococcus* spp (Fig. 6G) and a significant decrease in the abundance of *Pseudomonas* spp. (Fig. 6H) was observed in dsxiap/p47 RNA-injected compared to that in ds gfp-injected ticks.

### Transmission electron microscopic examination shows spirochetes within midgut epithelial cells poised to exit the tick midgut

**Fig. 7:**
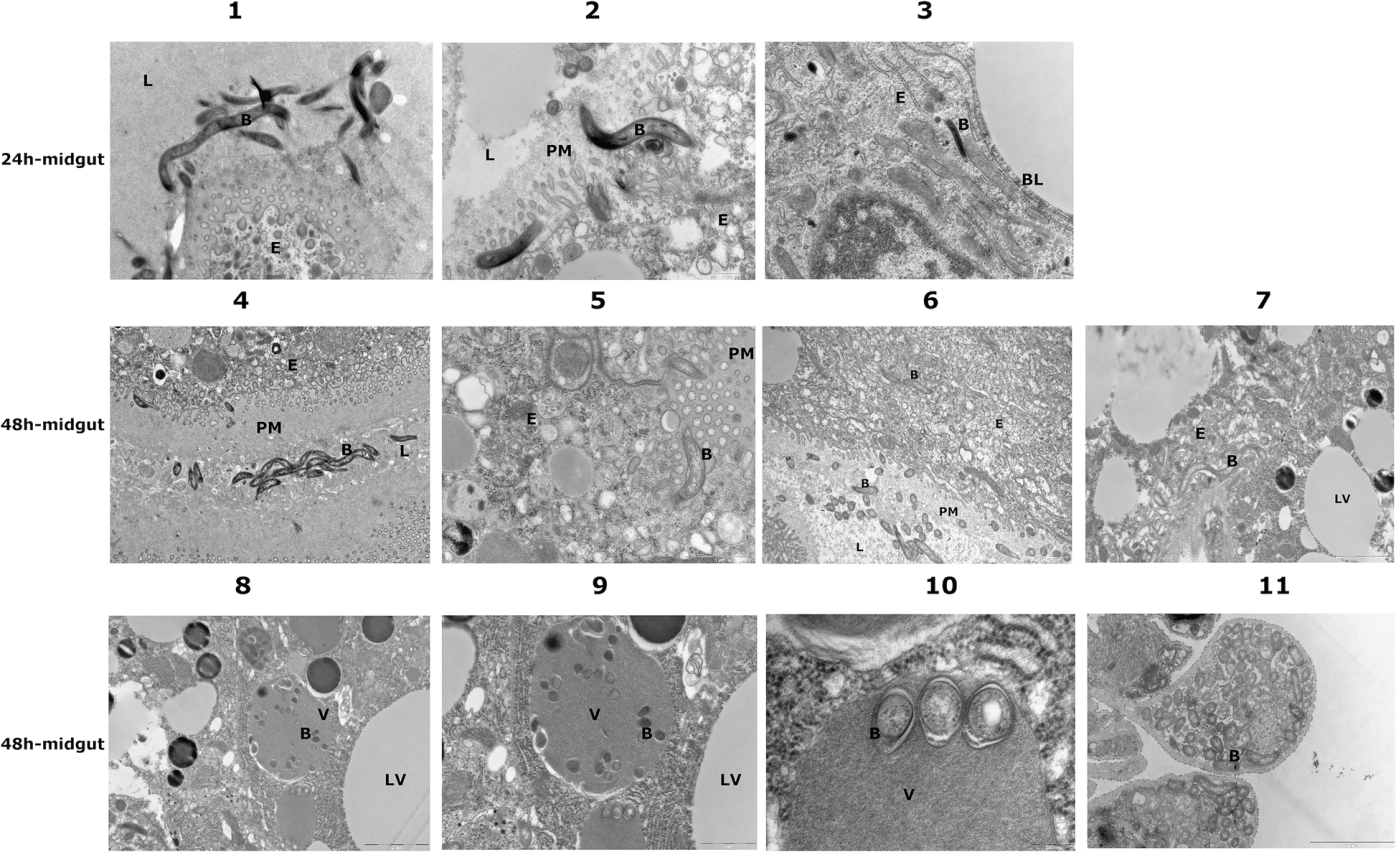
Transmission electron microscopy (TEM) of tick midguts shows intracellular *Borrelia burgdorferi* poised for egress*. B. burgdorferi*-infected ticks were fed on pathogen-free mice for 24 or 48 h, removed from the host, dissected to obtain tick midguts, processed for TEM analysis, and visualized. Panels 1, 2, 3, 4, 5, 6, and 7 are representative of ~10 midguts at 24 or 48 h of feeding showing *B. burgdorferi* in the midgut lumen, in the peritrophic matrix, and inside epithelial cells. Panels 8, 9, and 10 show *B. burgdorferi* inside vesicles adjacent to lipid vesicles and are shown at different magnifications for clarity. Panel 11 shows *B. burgdorferi*-containing ballooning extrusion of the midgut epithelial cell. B, *B. burgdorferi*; L, lumen; E, epithelial cells; BL, basal lamina; PM, peritrophic matrix; V, vesicles; LV, lipid vesicles

**Fig. 8:**
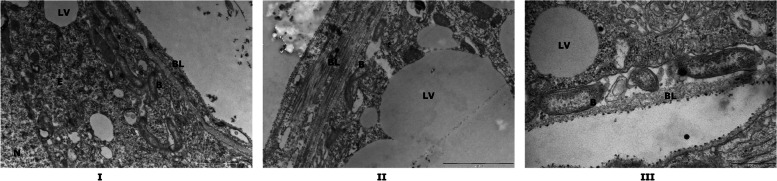
Transmission electron microscopy (TEM) of tick midguts shows intracellular *Borrelia burgdorferi* near the basal lamina of the midgut epithelial lining. *B. burgdorferi*-infected ticks fed on pathogen-free mice for 48 h were dissected to obtain tick midguts, processed for TEM analysis and visualized. Panels I, II, and III are representative of approximately 10 midguts at 48 h of feeding showing *B. burgdorferi* near the basal lamina, but no points of egress observed. B *B. burgdorferi*; E epithelial cells; BL basal lamina; LV, lipid vesicles

To begin to understand why the spirochete migration from the midgut was not influenced by alterations in the microbiome composition, we utilized transmission electron microscopy (TEM) to visualize *B. burgdorferi*-infected midguts at 24 and 48 h post-tick attachment to the host and midguts processed for microscopy as described in the “Materials and methods” section. While we observed spirochetes in proximity to the epithelial cells, in the peritrophic matrix and in the midgut lumen at 24 and at 48 h (Fig 7), we observed spirochetes within epithelial cells as early as 24 h post-tick attachment (Fig. 7). We also observed spirochetes in the vesicles inside epithelial cells and in ballooning midgut extrusions (Fig. 7). By 48 h post-tick attachment, we observed spirochetes lying close to the basal membrane of those epithelial cells (Fig. 8) in preparation for exit from the midgut, but none were observed to breach the basal barrier, suggesting that this is likely to be a rare event. At least 10–15 midguts/time points and 3 biological replicate experiments were performed.

## Discussion

The last decade has highlighted the need to understand the tick microbiome, including its nutritional endosymbionts, and the commensal bacteria that the tick must encounter during its on-host and off-host phase in the context of tick biology and vectorial competence [15, 33]. Earlier observations by Adegoke et al. [34] on *Hyalomma anatolicum* and *Rhipicephalus microplus* showed that infections with pathogens such as *Theileria* sp. had an impact on the tick microbiome composition. The increase in the relative abundance of *Acinetobacter* species in *B. burgdorferi*-infected nymphs compared to that in uninfected nymphs suggest that *B. burgdorferi* presence impacts the microbiome composition (Fig. 1). Whether *B. burgdorferi* plays a role in modulating tick innate immune responses that also surveil environmental microbiota that the tick encounters will need a thorough examination. *Acinetobacter* species are predominant Gram-negative soil microorganisms, and their association with ticks is not uncommon. However, there is currently no experimental or epidemiological demonstration of a potential role for *Acinetobacter* species in modulating tick-spirochete interactions. Robust approaches to mono-colonize ticks with specific commensal bacteria will be essential to determine the impact of these bacteria on tick biology and tick-pathogen interactions. A recent study by Oliver et al. [35] has shown that *I. scapularis* adults cured of their *R. buchneri* endosymbionts by antibiotic treatment on membrane feeding units are viable and offers an opportunity to understand the role of the nutritional endosymbionts in pathogen acquisition and transmission.

In this study, we examined the potential role of commensal bacterial microbiota that associate with the tick during their on- and off-host stages in modulating *B. burgdorferi* transmission to the mammalian host. Towards this, we sought to first generate ticks that would harbor no environmental bacteria by using germ-free isolators. Larvae raised in germ-free isolators or in normal incubators showed that the microbiome was predominantly composed of the nutritional endosymbiont, *R. buchneri*. In contrast, in our earlier work, we observed differences in the microbiome composition of larvae raised in sterile containers when compared to larvae raised in normal containers [16]. The use of stringent surface sterilization techniques including 10 % bleach, and 70 % ethanol, and the exclusion of all taxa represented at less than 1% relative abundance could account for this outcome. Subsequent to feeding, the composition of the microbiome of GF larvae was shown to be different when compared to that of normal larvae, potentially due to exposure of normal larvae to commensal bacteria on the normal murine skin (Fig. 1). The use of sterile or normal containers used in our previous study to raise larvae [16] provided a differential exposure of ticks to environmental bacteria. We observed that differential exposure impacts *B. burgdorferi* acquisition and that sterile-container-raised larvae acquired *B. burgdorferi* less efficiently compared to larvae raised in normal containers [16]. Germ-free isolators used in the current study are devoid of environmental bacteria. Therefore, GF-isolator-raised larvae in this study are exposed only to *B. burgdorferi* during acquisition. Although larvae raised in GF or normal isolators acquired *B. burgdorferi* comparably, *B. burgdorferi* in GF-raised larvae were sustained more efficiently during the molt to nymphs when compared to that in normal incubator-raised larvae (Fig. 2). We speculate that in the absence of exposure to environmental bacteria, the gut lumen is more hospitable to *B. burgdorferi* survival. Differences in the surface sterilization methods and batch differences in ticks between the two studies preclude a direct comparison. Nonetheless, these observations collectively suggest that changes in exposure to environmental microbiota do have an impact on *B. burgdorferi* acquisition, colonization, or survival during larval molting, in consistent with our earlier observations [16, 20]. We also note that the GF-raised ticks were not devoid of bacteria. Since mated adult females were only surface sterilized prior to placement into the GF isolators, bacteria associated with the adult tick gut or other tissues are not eliminated. Membrane feeding of adult ticks with combinations of antibiotics may circumvent this limitation in future studies.

Nymphs that molted in GF isolators showed changes in the microbiota composition when compared to normal isolator-raised nymphs especially upon feeding on the murine host (Supplemental Fig 1). Nevertheless, GF-isolator-raised *B. burgdorferi*-infected nymphs, despite harboring a higher burden of spirochetes than normal incubator-raised *B. burgdorferi*-infected nymphs, showed comparable spirochete growth in the midguts and migration to salivary glands and transmitted spirochetes comparably to the murine host (Fig. 3). To rule out the possibility that our observation was not due to any microbiome unrelated impact on GF-isolator-raised ticks, we altered the microbiome composition using additional strategies. Perturbation of the microbiome by feeding gentamicin-resistant *B. burgdorferi*-infected ticks on gentamicin-injected mice also demonstrated a redundant role for the tick microbiome during spirochete transmission (Fig. 4). Despite a significant reduction in total bacterial burden in gentamicin exposed nymphs, we observed only modest reduction in predominant tick-associated bacterial genera such as *Staphylococcus* spp. and no impact on *Pseudomonas* spp. A global microbiome sequencing may be more insightful. These observations suggested that *B. burgdorferi* migration from the midgut to the salivary glands and transmission to the host was not significantly modulated by environment-acquired microbiota.

Direct perturbation of the gut microbiota composition by the introduction of wild-type *Pseudomonas aeruginosa* PA103, as well as the type 3 secretion mutant PA103*exs*E, a type 3 secretion system mutant [36] by analpore injection, diminished survival significantly and could not be analyzed further. While the introduction of *Staphylococcus aureus* SA113 into *B. burgdorferi*-infected tick midgut was less deleterious to tick survival, the yield of viable ticks was low and was not amenable to detailed analyses. Future studies may utilize non-pathogenic *Staphylococcus* species that are normal skin commensals. We acknowledge that the anal pore route is a non-natural route and introduction by the oral route should be assessed. However, introduction by artificial membrane feeding was not viable since maintaining the membranes sterile during tick feeding required the addition of antibiotics and confounded our ability to introduce these bacteria.

STAT, the central transcription factor of this pathway regulates the expression of multiple effector genes involved in repair, remodeling, and immunity [37, 38]. Therefore, we expected that RNAi-mediated knockdown of *stat* would have an impact on the microbiome. Indeed, *stat* knockdown ticks showed differences in the microbiome composition based on the qPCR assessment of specific commensal bacteria. Nevertheless, changes in the microbiome composition did not influence *B. burgdorferi* growth in the midgut, invasion of salivary glands, or transmission to the vertebrate host (Fig. 5). Our earlier work suggested that STAT regulates the expression of Peritrophin-1 a key component of the peritrophic matrix (PM) and that integrity of the PM ensures survival and *B. burgdorferi* colonization of the tick gut [16]. In this study, while *stat* knockdown resulted in decreased expression levels of *peritrophin-1* and diffuse PM as seen by TEM visualization—there was no impact on *B. burgdorferi* egress from the gut and transmission to the mammalian host.

We do not have a mechanistic understanding of the effect of stat-knockdown on increasing *R. buchneri* (an intracellular Gram-negative bacterium) abundance and decreasing *Staphylococcus* spp. (extracellular Gram-positive bacteria) abundance. The tick immunome is not fully understood, and whether there is cross-talk between JAK/STAT pathway and other immune pathways is also not known [39]. Interfering with the IMD pathway [31] by silencing key components of this pathway also resulted in changes in the abundance of *Staphylococcus* spp. and *Pseudomonas* spp. by mechanisms that remain to be deciphered. A better understanding of the tick immunome may help clarify the differential impact of the JAK/STAT and IMD pathways on the tick microbiome. While we observed an increase in *B. burgdorferi* burden in the midgut upon interrupting the IMD pathway, there was no impact on migration to salivary glands (Fig. 6). It is likely that IMD-mediated signaling of effectors such as antimicrobial peptides (AMP) may impact *B. burgdorferi* survival in the midgut. AMPs stimulated by the IMD pathways remain to be characterized and will be critical to better understand this aspect of *B. burgdorferi*-IMD pathway interactions.

Spirochetes enter the tick midgut along with the bloodmeal and during colonization of the tick are vulnerable to the direct or indirect impact of midgut microbiota and other detrimental luminal components [16, 40]. In contrast, modulating the exposure of ticks to environmental microbiota does not impact spirochete transmission to the murine host. We reasoned that the spirochete may use a unique strategy to escape the impact of commensal microbiota during this event. A live imaging study by Dunham-Ems et al. [26] utilized confocal imaging to examine spirochete egress from the gut and suggested a biphasic mode of spirochete migration from the tick—a first non-motile phase composed of a network of spirochetes replicating intercellularly towards the basal lamina in the first 48 h of tick feeding, followed by a motile phase around 72h of tick feeding, also potentially intercellularly between gaps in tight junctions. Since the magnification achieved by TEM allows more cellular clarity, we utilized TEM imaging to begin to understand why changes in exposure to environmental microbiota have no significant impact on spirochete egress from the tick midgut. Our observations using transmission electron microscopy suggest that while some spirochetes are sequestered between the peritrophic matrix and the epithelial cells some spirochetes also are intracellular, entering midgut epithelial cells as early as 24 h post-tick attachment to the mammalian host. These observations suggest that some spirochetes may enter the midgut epithelial cells within 24 h post-attachment in preparation for migration to the salivary glands and potentially escape any challenges presented by microbiota in the luminal side of the midgut epithelial cells. Zung et al. [41] utilized TEM almost three decades ago and also suggested a potential intracellular phase of the spirochetes during transmission. Interestingly, Zung et al. [41] also observed spirochetes intercellularly with 4–5 h of tick feeding—a time point that is not thought to be coincident with spirochete transmission. We do not rule out the potential intercellular route suggested by earlier studies. In our studies, we observed that the epithelial cells for the most part were tightly apposed to each other, and we rarely observed spirochetes between epithelial cells (in one instance out of 10–15 guts examined) (Supplemental Figure 3). We also observed spirochetes in what appear to be vesicles. Whether these represent lipid vesicles remains to be determined. The spirochete-filled ballooning extrusions in midgut epithelial cells were also suggestive of unique cellular events that may enable migration from the gut. While we observed spirochetes poised to exit the basal lamina of the midgut, we could not observe the point of exit (Fig. 8). We have conducted TEM on 48-h fed midguts (10–15 ticks and 3 biological replicates), a timepoint that marks active egress from the midgut. Dunham-Ems et al. [26] have noted that spirochete migration through the basal lamina was a rare event even at 72h of tick feeding. Optimizing the EM process to accommodate replete tick midguts may clarify the route of egress further. A recent study by Pospisilova et al. [42] suggests that *B. afzelii* might be transmitted to the murine host via regurgitation of spirochetes in the midgut lumen. Such a route of transmission has not been evidenced for *B. burgdorferi* [43].

*B. burgdorferi* is an extracellular organism [44], and our observations are not to be construed as contesting this dogma. Spirochete migration from the midgut to the salivary glands in the tick vector and from blood vessels across the endothelium in the mammalian host is critical for transmission to the vertebrate host and dissemination in the mammalian host, and we understand very little of these key events. It has been suggested that some spirochetes likely transmigrate through the endothelial cells and that *B. burgdorferi* molecules such as P66 and BBK32 may play a dominant role in this event [45, 46]. Spirochete migration across the blood-brain barrier results in neuroborreliosis [47], and this must also call for unique navigation strategies that are not understood. It is likely that *B. burgdorferi*, traditionally an extracellular pathogen, utilizes an intracellular route, albeit transient, during certain key events in its life cycle. A mechanistic understanding of this intracellular transit may reveal fundamental insights into spirochete biology and its complex life cycle.

## Conclusions

Our findings suggest a redundant role for environment-acquired tick microbiota during spirochete transmission from the tick to the mammalian host. The findings also highlight an intracellular phase of the spirochete in the tick midgut that may allow the spirochete to circumvent interactions with environment-associated microbiota during transmission. The study sets the stage for detailed molecular studies into this facet of tick-spirochete interactions, a less-understood, albeit key determinant of transmission to the vertebrate host.

## Materials and methods

### Ethics statement for animal use

Animal care and housing followed the rules described in the Guide for the Care and Use of Laboratory Animals of the National Institutes of Health, USA. The protocols described below for the use of mice were reviewed and approved by the Yale University Institutional Animal Care and Use Committee (YUIACUC), and the approved Protocol number is 2020-07941. All animal experiments were conducted in a Biosafety Level 2 animal facility according to YUIACUC rules.

### Generation of ticks in germ-free isolators to generate dysbiosed ticks

*I. scapularis* adults were obtained from a tick colony at the Oklahoma State University, Stillwater, OK, and maintained in an incubator at 23°C and 85% relative humidity under a 14-h light and 10-h dark photoperiod. Female New Zealand white rabbits (Charles River, MA) were used to feed female adult ticks essentially as described earlier [48]. The fed adult ticks were surface sterilized in 10 % bleach for 15 min, sterile 70 % ethanol for 30 min, and then aseptically introduced into a germ-free isolator (Park Bioservices, MA) available at the Yale Microbial Diversity Institute, West Campus, Yale University, and adults allowed to lay eggs in a desiccator provided with sterile saturated magnesium sulfate (relative humidity ~85%, temperature 23°C and light/dark 14:10). The eggs then hatched into larvae in the germ-free isolator. Ticks harbor several bacteria that might be acquired vertically from the mother and these would likely not be eliminated using this approach. However, bacteria acquired from the environment (allochthonic) would be absent in the germ-free setting, and these larvae will be dysbiosed relative to ticks reared in a normal incubator and referred to as GF-reared larvae. Control adults were surface-sterilized and placed in normal incubators maintained under the same humidity, temperature, and light/dark cycle as described above and egg laying and hatching were allowed to hatch in normal incubators.

Germ-free (GF) mice (C57/BL6, Taconic Farms) were needle-inoculated with *B. burgdorferi* (N40) intradermally and infection was confirmed by quantitative (q)PCR of skin punch biopsy at day 21 post-infection as described earlier [16] and comparably infected mice utilized for colonization studies. GF-reared larvae were then fed to repletion on *B. burgdorferi*-infected GF mice (~ 150 larvae/mouse). To assess *B. burgdorferi* colonization, the total DNA was isolated from pools of 5 larvae using the DNeasy blood and tissue kit (Qiagen) and *B. burgdorferi* burden was assessed in about 50 larvae by qPCR as described earlier [16].

To assess nymphal stages, repleted larvae were collected and placed in sterile/pyrogen-free tubes and allowed to molt to GF-reared-nymphs. All manipulations were done in the germ-free isolator strictly adhering to germ-free and BL2 procedures. Control larvae and nymphs were similarly generated in parallel in normal incubators maintained at the same temperature, humidity, and light/dark cycle as above and larvae fed on specific pathogen-free age-matched female C57/BL6 mice (Taconic Farms) and infected with *B. burgdorferi* (N40) as described above.

#### Dysbiosis by antibiotic treatment

Transgenic *B. burgdorferi*: 297 strain carrying a gentamicin antibiotic resistance cassette and a green fluorescent protein marker inserted into the highly stable cp26 plasmid [26], Bb914, referred to henceforth as Bb-GFP, were utilized to infect 4–5-week-old C3H/HeN mice obtained from Charles River Laboratories as described earlier [16]. *I. scapularis* larvae obtained from the Oklahoma State University, Stillwater, OK, were fed to repletion on Bb-GFP-infected mice and larvae were allowed to molt over 6–8 weeks in a normal incubator maintained as described above. Midguts from 15 to 20 molted flat nymphs were dissected individually, and *B. burgdorferi* burden was assessed by qPCR as described earlier [16]. For perturbing the microbiome using gentamicin, 5 female C3H/HeN mice were injected intraperitoneally with 100 μL of 10 mg/mL gentamicin in PBS 24 h prior to and once on the day of tick placement. Control mice received PBS in the same manner.

### Tick RNA, DNA isolation, and quantitative PCR

Unfed larval ticks were ground in liquid nitrogen in pools of 50, or fed larval ticks were ground in pools of 5 and suspended in DNAeasy lysis buffer for DNA preparation using the DNeasy genomic DNA preparation kit (Qiagen, CA). Nymphal ticks fed to repletion on the murine host were dissected and salivary glands and midguts pooled (3 ticks) and homogenized and the DNA was extracted as described above. The same procedure was performed to assess the midguts of unfed ticks, when warranted. For all bacterial microbiota burden assessment *16S rRNA* (*universal or genera-specific*), *Rickettsia buchneri*, *Staphylococcus* genera-specific, and *Pseudomonas* genera-specifc 16S rRNA primers were utilized as described earlier [20] and the DNA was used directly for quantitative PCR (qPCR) and data normalized to tick actin using tick actin primers described earlier [16]. In RNA interference experiments, the midgut and salivary gland tissues of repleted ticks were suspended in 200 μL of Trizol (Invitrogen, CA), and the total RNA was prepared according to the manufacturer’s protocol. cDNA was synthesized from the total RNA using the iScript RT-PCR kit (Biorad, CA) and analyzed by quantitative RT-PCR for the expression of specific gene transcripts including tick actin*, stat, p47, xiap,* and *peritrophin1 (prtf1)* using the iQ Sybr Green Supermix (Biorad, CA) on a MJ cycler (MJ Research, CA) and primers as described earlier [16, 32]. In RNAi experiments, a subset of nymphal ticks was also processed for total DNA extraction as described above. *B. burgdorferi* burden was assessed by qPCR in DNA samples using *B. burgdorferi*-specific *flaB* primers described earlier [20], and microbiota burden was assessed by qPCR as described above. The data was normalized to tick actin in qPCR and qRT-PCR assays.

### RNAi silencing in *B. burgdorferi*-infected *I. scapularis* nymphs

dsRNA complementary to *stat* was synthesized by using the MEGAscript RNAi kit, and dsRNA was purified and quantified spectroscopically (Ambion) as described earlier and dsRNA (40 nL) injected into the anal pore of *B. burgdorferi*-infected nymphs using 10-μl microdispensers (Drummond Scientific, Broomall, PA) [16]. Control nymphs were injected with an irrelevant dsRNA complementary to GFP [16]. Si (small interfering) RNA for xiap and p47 was generated as described earlier [32] and mixed at equimolar amounts and injected into the anal pore of *B. burgdorferi*-infected nymphs as described above. Control nymphs were similarly injected with scrambled RNA prepared as described earlier [32].

### Microbiome composition analysis

The microbiome compositions of ticks raised in GF isolators or normal incubators were assessed at the larval and nymphal stage by amplifying the V4 regions of the bacterial 16*S* rRNA using protocols outlined by the Earth Microbiome Project. DNA from unfed nymphs (in pools of 5 tick midguts) and fed nymphs (15–20 individual nymphal midguts) were prepared and processed for 16S rRNA amplicon preparation and sequencing using the 454 sequencers as described earlier [16]. DNA from unfed or fed larvae was prepared as described earlier [20] (pooling about 50 unfed larvae or 5 fed larvae to assess up to 500 or 50 larvae, respectively). Bacterial 16S rDNA amplicons were generated using barcoded 16S universal primers (515F/806R) as outlined by the Earth Microbiome Project (www.earthmicrobiome.org). The amplicons were purified using the PCR purification kit (Qiagen), and equimolar amounts of each were pooled prior to sequencing on an Illumina MiSeq system. The 454 sequence data of the microbiota in the nymphal samples were analyzed using the QIIME1 version 1.9.1 [49]. The operational taxonomic units (OTUs) were generated using the QIIME1 pick_open_reference_otus.py script with usearch as the search engine [50]. The microbiota in the larvae samples were analyzed using the QIIME2, version 2020.11, [51] custom pipeline. The paired-end reads from MiSeq (2 x 250 bp) were demultiplexed, trimmed, quality-filtered, and joined in QIIME2. The sequences were then denoised, and OTUs were generated using Deblur [52]. The generated OTUs were assigned to taxonomy using a pre-trained Naïve Bayes classifier on the V3–V4 regions of the 16S rRNA sequences from the Greengenes database, release 13.8 [53] at 99% similarity. The group differences between the normal and GF-raised larval samples were calculated using ANOSIM, using the weighted UniFrac distances. Only bacterial genera represented at >/=1 % relative abundance in tick samples were included in the analysis. OTUs that were represented in the water samples at relative abundances comparable to that in the experimental samples represented non-specific contaminants inherent during the processing of samples and were not included in the analysis.

### *B. burgdorferi* transmission to the murine host

In all transmission experiments, at least 4 *B. burgdorferi*-infected nymphs were placed on each mouse (5 mice used in each control or experimental group) and ticks were allowed to feed to engorgement, weighed, and midguts and salivary glands (SG) dissected for genomic DNA purification and *B. burgdorferi flaB* amplicons assessed by qPCR as described above. In transmission experiments, utilizing germ-free isolator-reared experimental and control ticks of C57/BL6 mice was used. For all other transmission experiments, C3H/HeN mice were utilized. All experiments were replicated three times. In RNA interference experiments, the total RNA was isolated using trizol (Thermo Fisher Scientific) to confirm gene silencing, and *B. burgdorferi flaB* transcripts were simultaneously assessed as described [20]. *B. burgdorferi* burden in mice 5, 7 or 10 (representing early time post tick bite), and 14 and 21 days post-engorgement was also assessed by purifying DNA from the tissues (skin and in the heart and joints) using the DNeasy blood and tissue kit (Qiagen) and *flaB* amplicons assessed by qPCR and normalized to mouse actin [20].

### Transmission electron microscopy of tick midguts

*B. burgdorferi-*infected *I. scapularis* nymphs were allowed to feed on C3H/HeN mice for 24 or 48 h. The ticks were then carefully detached from the mice and dissected in 2 % paraformaldehyde (PFA) in PBS and 2.5 % glutaraldehyde. The midguts were gently rinsed and placed in 2 % PFA and 2.5 % glutaraldehyde containing 0.05 % Ruthidium red for half an hour at room temperature and half an hour at 4°C, rinsed in PBS, dehydrated in an ethanol series, embedded in epoxy resin, hardened, and ultra-thin sectioned for transmission electron microscopic visualization (TEM) on a FEI Tencai Biotwin transmission electron microscope at 80Kv. Images were taken using Morada CCD and iTEM (Olympus) software. At least 10–15 ticks were utilized for each time point, and three replicate experiments were performed.

### Statistical analysis

The significance of the difference between the mean values of the control and experimental groups in quantitative PCR assays was analyzed using the Mann-Whitney *U* test, and when more than two groups were assessed, the statistical significance was assessed using ANOVA with Tukey’s or Dunn’s multiple comparisons test using Prism 7.0 software (GraphPad Software, USA). *p* ≤ 0.05 was considered statistically significant. Primers for tick genes were designed using OligoPerfect^TM^ Designer (Thermoscientific).

## Supplementary Information

**Additional file 1: Supplementary Figure 1**. Abundance of predominant microbiota associated with *I. scapularis* nymphs raised in germ-free isolators or normal incubators. *B. burgdorferi-*infected *I. scapularis* nymphs raised in germ-free isolators (GF) or normal incubators and fed to repletion on germ-free or normal pathogen-free C57/BL6 respectively. Abundance of predominant microbiota was assessed in the midguts of unfed (A-D) and fed (E-H) GF and normal nymphs by qPCR using universal 16S primers (A and E); primers targeting 16S rRNA specific to *Rickettsia buchneri* (B and F), *Staphylococcus genera* (C and G)*; Pseudomonas* genera (D and H). Each data point in represents a pool of 3 tick midguts; Error bars are + SEM. Significance of differences assessed by non-parametric Mann-Whitney test (**p<0.005, ***p<0.0001).

**Additional file 2: Supplementary Figure 2.** Changes in microbiota composition in *stat* knockdown nymphs. Double stranded *stat* RNA (ds stat) or control ds gfp RNA (ds gfp) was injected into the anal pore of *B. burgdorferi*-infected nymphs and ticks fed to repletion on pathogen-free C3H/HeN mice and the abundance of specific bacteria assessed by qPCR. A. Total bacterial burden based on amounts of 16S rRNA as a proxy for total bacterial burden; B. *Rickettsia buchneri (R. buchneri)-*specific 16S rRNA amplicons*;* C. *Staphylococcus genera-*specific 16S rRNA amplicons; and D *Pseudomonas* genera-specific 16S rRNA amplicons. Each data point represents a pool of 3-4 tick midguts; Error bars are + SEM. Significance of differences assessed by non-parametric Mann-Whitney test ( *p<0.05).

**Additional file 3: Supplementary Figure 3.** Transmission electron microscopy (TEM) of tick midguts shows apposed tight junctions*. B. burgdorferi*-infected ticks were fed on pathogen-free mice for 48 h, removed from the host, dissected to obtain tick midguts, processed for TEM analysis and visualized. Panels I, and II are representative of approximately 10 midguts at 48 h of feeding showing *B. burgdorferi* in the midgut lumen and the tight junctions between two adjacent epithelial cells. Panel III is one of approximately 10 midguts at 48 h of feeding showing *B. burgdorferi* between the tight junctions of two epithelial cells B, *B. burgdorferi;* L, lumen*;* E, epithelial cells; PM, peritrophic matrix. Arrows indicate the tight junctions.

## Data Availability

All data generated in this work will be readily shared and available upon request. The sequence data has been deposited in the NCBI Biorepository database. The accession number for the BioProject is PRJNA232504.

## Abbreviations

AMP: Antimicrobial peptide
cDNA: Complementary DNA
dsRNA: Double-stranded RNA
*flaB*: *FlagellinB*
GF: Germ-free
GFP: Green fluorescent protein
IMD: Immune deficiency
JAK/STAT: Janus kinase/signal transducer and activator of transcription
PM: Peritrophic matrix
*prtf1*: Peritrophin1
PCR: Polymerase chain reaction
PCoA: Principal Coordinate Analysis
qPCR: Quantitative PCR
qRT-PCR: Quantitative reverse transcriptase-PCR
rRNA: Ribosomal RNA
siRNA: Small interfering RNA
TEM: Transmission electron microscopy
XIAP: X-linked inhibitor of apoptosis
