# Supplementary figures and images for: Tick transmission of Borrelia burgdorferi to the murine host is not influenced by environmentally acquired midgut microbiota
Source: Microbiome. 2022 Oct 17;10:173. doi: 10.1186/s40168-022-01378-w (PMC9575305; doi:10.1186/s40168-022-01378-w)

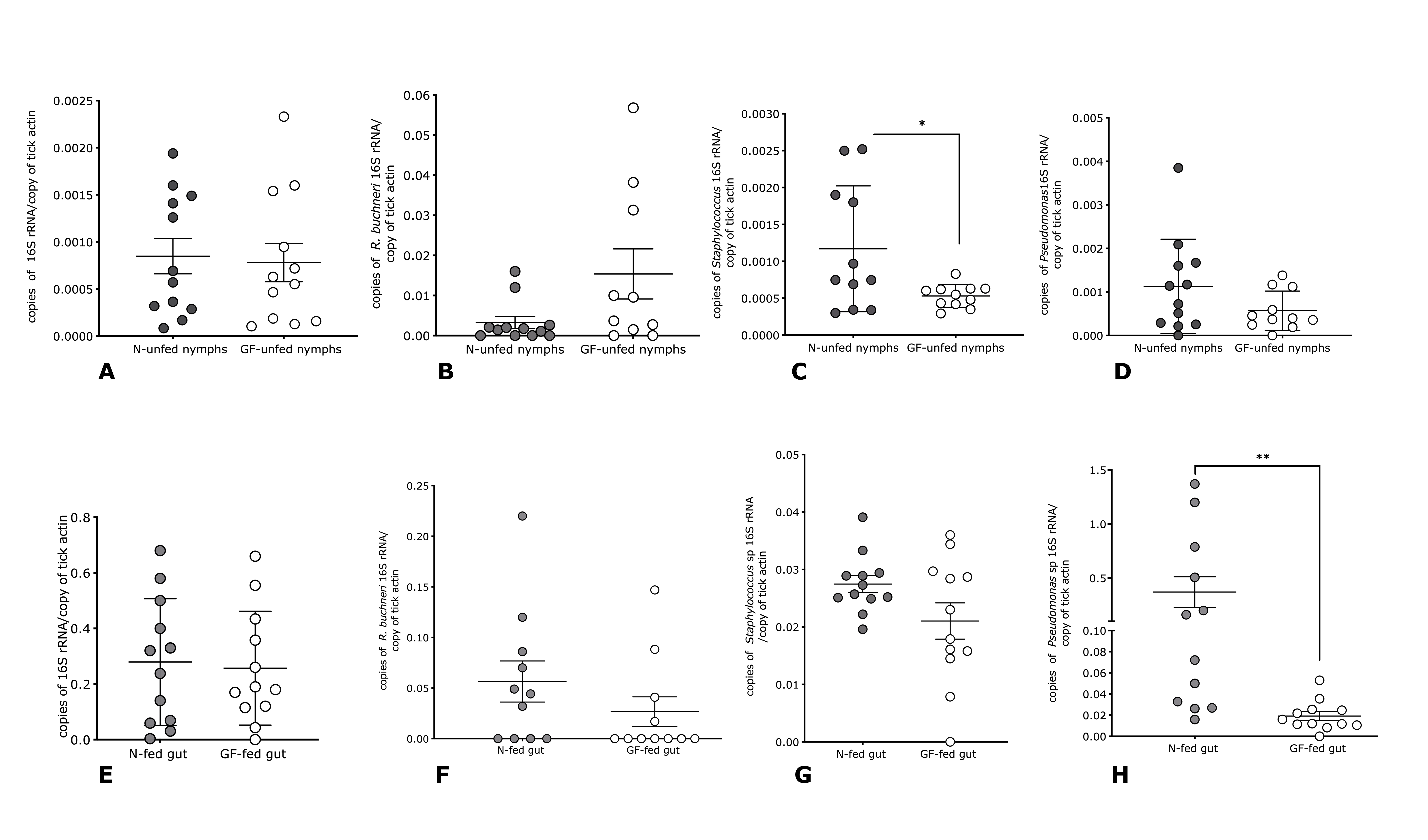

Supplement: Supplementary file 2 — Additional file 1: Supplementary Figure 1. Abundance of predominant microbiota associated with I. scapularis nymphs raised in germ-free isolators or normal incubators. B. burgdorferi-infected I. scapularis nymphs raised in germ-free isolators (GF) or normal incubators and fed to repletion on germ-free or normal pathogen-free C57/BL6 respectively. Abundance of predominant microbiota was assessed in the midguts of unfed (A-D) and fed (E-H) GF and normal nymphs by qPCR using universal 16S primers (A and E); primers targeting 16S rRNA specific to Rickettsia buchneri (B and F), Staphylococcus genera (C and G); Pseudomonas genera (D and H). Each data point in represents a pool of 3 tick midguts; Error bars are + SEM. Significance of differences assessed by non-parametric Mann-Whitney test (**p<0.005, ***p<0.0001). [file 40168_2022_1378_MOESM1_ESM.tif]

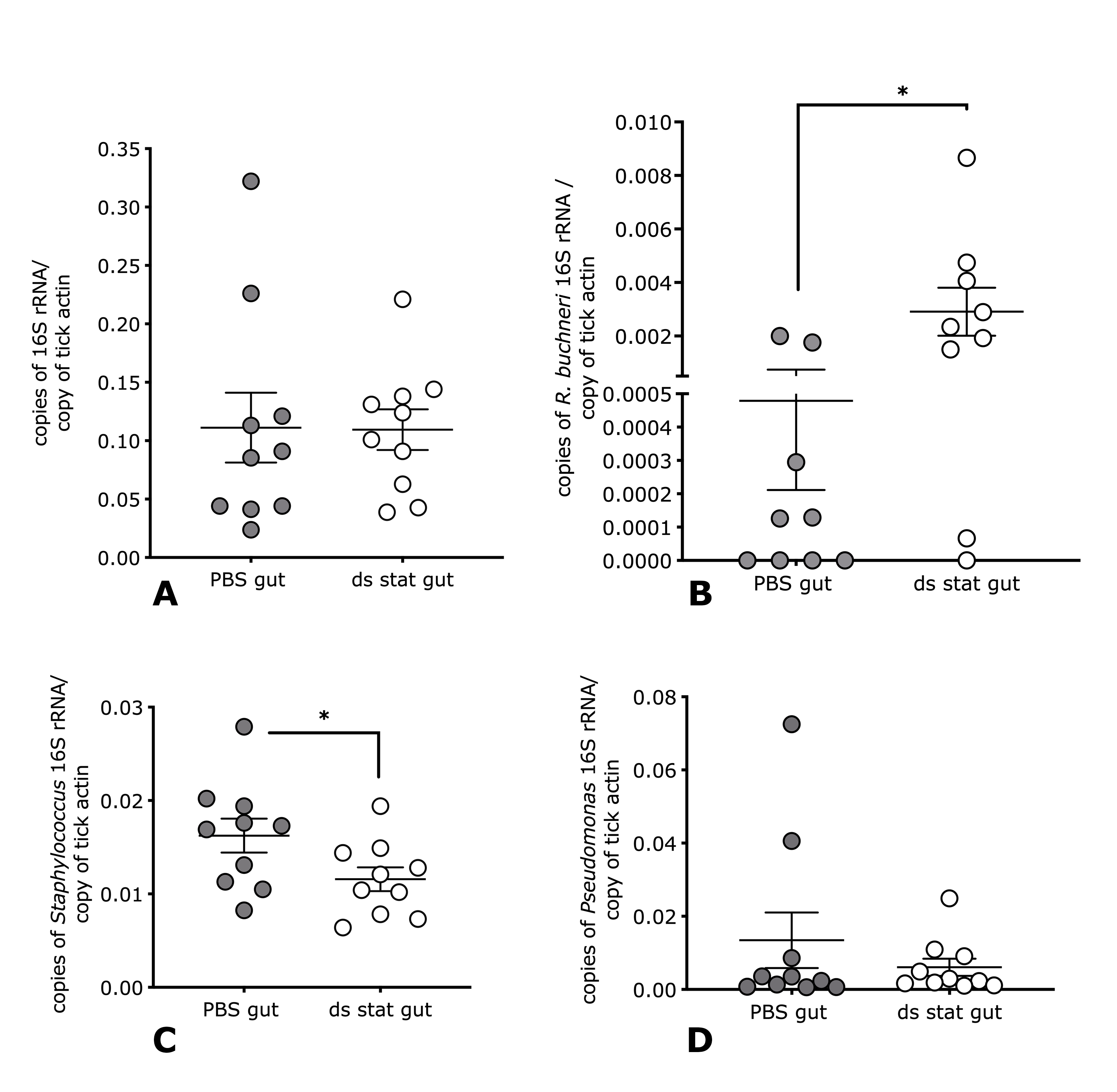

Supplement: Supplementary file 3 — Additional file 2: Supplementary Figure 2. Changes in microbiota composition in stat knockdown nymphs. Double stranded stat RNA (ds stat) or control ds gfp RNA (ds gfp) was injected into the anal pore of B. burgdorferi-infected nymphs and ticks fed to repletion on pathogen-free C3H/HeN mice and the abundance of specific bacteria assessed by qPCR. A. Total bacterial burden based on amounts of 16S rRNA as a proxy for total bacterial burden; B. Rickettsia buchneri (R. buchneri)-specific 16S rRNA amplicons; C. Staphylococcus genera-specific 16S rRNA amplicons; and D Pseudomonas genera-specific 16S rRNA amplicons. Each data point represents a pool of 3-4 tick midguts; Error bars are + SEM. Significance of differences assessed by non-parametric Mann-Whitney test ( *p<0.05). [file 40168_2022_1378_MOESM2_ESM.tif]

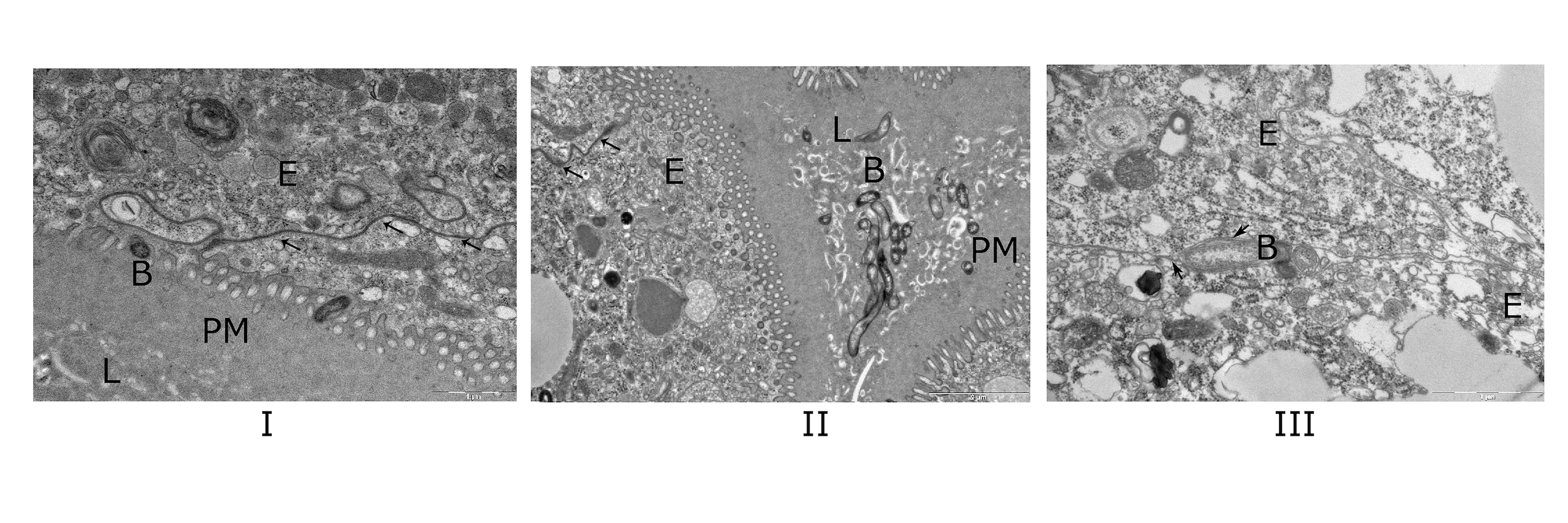

Supplement: Supplementary file 4 — Additional file 3: Supplementary Figure 3. Transmission electron microscopy (TEM) of tick midguts shows apposed tight junctions. B. burgdorferi-infected ticks were fed on pathogen-free mice for 48 h, removed from the host, dissected to obtain tick midguts, processed for TEM analysis and visualized. Panels I, and II are representative of approximately 10 midguts at 48 h of feeding showing B. burgdorferi in the midgut lumen and the tight junctions between two adjacent epithelial cells. Panel III is one of approximately 10 midguts at 48 h of feeding showing B. burgdorferi between the tight junctions of two epithelial cells B, B. burgdorferi; L, lumen; E, epithelial cells; PM, peritrophic matrix. Arrows indicate the tight junctions. [file 40168_2022_1378_MOESM3_ESM.tif]
